# Baseline staging tests based on molecular subtype is necessary for newly diagnosed breast cancer

**DOI:** 10.1186/1756-9966-33-28

**Published:** 2014-03-17

**Authors:** Xuesong Chen, Lichun Sun, Yingying Cong, Tingting Zhang, Qiushi Lin, Qingwei Meng, Hui Pang, Yanbin Zhao, Yu Li, Li Cai, Xiaoqun Dong

**Affiliations:** 1 Department of Internal Medical Oncology, Harbin Medical University Cancer Hospital, Harbin, Heilongjiang Province 150040, China; 2 Department of Internal Medical Oncology, the Tumor Hospital of Jilin Province, Changchun, Jilin Province, China; 3 Department of Biomedical and Pharmaceutical Sciences, College of Pharmacy, University of Rhode Island, Pharmacy Building, 7 Greenhouse Road, Kingston, RI 02881, USA; 4 Bacteriologic Laboratory, Harbin Center for Disease Control and Prevention, Harbin, Heilongjiang Province, China

**Keywords:** Molecular subtype, Baseline staging, Breast cancer

## Abstract

**Background:**

Bone scanning (BS), liver ultrasonography (LUS), and chest radiography (CXR) are commonly recommended for baseline staging in patients with newly diagnosed breast cancer. The purpose of this study is to demonstrate whether these tests are indicated for specific patient subpopulation based on clinical staging and molecular subtype.

**Methods:**

A retrospective study on 5406 patients with newly diagnosed breast cancer was conducted to identify differences in occurrence of metastasis based on clinical staging and molecular subtypes. All patients had been evaluated by BS, LUS and CXR at diagnosis.

**Results:**

Complete information on clinical staging was available in 5184 patients. For stage I, II, and III, bone metastasis rate was 0%, 0.6% and 2.7%, respectively (*P* < 0.01); liver metastasis rate was 0%, 0.1%, and 1.0%, respectively (*P* < 0.01); lung metastasis rate was 0.1%, 0.1%, and 0.7%, respectively (*P* < 0.01). Complete information on molecular subtype was available in 3411 patients. For Luminal A, Luminal B (HER2^-^), Luminal BH (HER2^+^), HER2^+^ overexpression, and Basal-like, bone metastasis rate was 1.4%, 0.7%, 2.5%, 2.7%, and 0.9%, respectively (*P* < 0.05); liver metastasis rate was 0.1%, 0.1%, 1.0%, 1.1%, and 0.9%, respectively (*P* < 0.01); lung metastasis rate was 0.20%, 0%, 0%, 0.27%, and 0.9%, respectively (*P* < 0.05). cT (tumor size), cN (lymph node), PR (progesterone receptor), and HER2 status predicted bone metastasis (*P* < 0.05). cT, cN, ER (estrogen receptor), PR, and HER2 status predicted liver metastasis (*P* < 0.05). cT, cN, and PR status predicted lung metastasis (*P* < 0.05).

**Conclusion:**

These data indicate that based on clinical staging and molecular subtypes, BS, LUS and CXR are necessary for patients with newly diagnosed breast cancer.

## Introduction

Breast cancer is the most common malignancy in women in the world and patients are assigned a clinical stage at diagnosis for efficient local and systemic treatment. It has been shown that detectable metastatic disease is a low probability event increased with clinical staging in the newly diagnosed breast cancer [1,2]. Most common metastatic sites include bone, lung, and liver. Previous studies proposed unnecessary examinations without affecting the efficacy of diagnosis and treatment [3,4], in order to save health expenditure and provide optimal use of resources.

To better understand molecular pathogenesis of breast cancer, immunohistochemistry and cDNA microarray have been used to define major subtypes based on receptor status [5,6]. Based on the presence of estrogen receptors (ER), progesterone receptors (PR) and human epidermal growth factor receptor 2 (HER2, also known as Neu/ErbB-2), receptor status along with tumor grading has categorized breast cancer into several conceptual molecular classes [7,8]. The 12th St Gallen International Breast Cancer Conference (2011) Expert Panel adopted a new approach to the classification of patients for therapeutic purposes. Proposed molecular subtypes [9-11] include: 1) Luminal A [12,13]: ER^+^ and/or PR^+^, HER2^-^, Ki67 < 14%; 2) Luminal B: ER^+^ and/or PR^+^, HER2^-^, Ki67 ≥ 14%; 3) Luminal BH: ER^+^ and/or PR^+^, any Ki67, HER2 overexpressed or amplified; 4) ERBB2/HER2 overexpression: ER^-^/PR^-^, HER2/neu overexpressed or amplified; [14] and 5) Basal-like [15]: ER^-^/PR^-^/HER2^-^ (also called triple negative breast cancer, TNBC; most BRCA1-mutant breast cancers are basal-like TNBC) [16]. These molecular subtypes are characterized by different epidemiological risk factors, tumor progression processes, responses to therapy and prognosis [17]. Baseline staging procedures such as bone scanning (BS), liver ultrasonography (LUS), and chest radiography (CXR) have been applied in clinical trials of adjuvant therapy of breast cancer. The current international guidelines for the management of breast cancer are generally against routine use of the above three examinations to detect asymptomatic distant metastases in patients with newly diagnosed early-staged breast cancer [4,18-20]. However, whether specific patient subpopulation based on clinical staging and molecular subtype would benefit from the above examinations needs further investigation. Therefore, the aim of this study was to determine the frequency and distribution pattern of metastases categorized by clinical staging and molecular subtypes in newly diagnosed breast cancer patients and to explore the valuable prognosis factors for bone, liver, and lung metastasis.

## Materials and methods

5406 patients with newly diagnosed breast cancer from January 2000 to July 2010 at Harbin Medical University Cancer Hospital were included in the current study. The median age of the patients was 60 (ranging from 18 to 75). 102 patients with bone pain symptoms or elevated transaminase or alkaline phosphatase and 120 patients with inadequate information of clinical stage were excluded, leaving 5184 cases for further analysis. Among them 3411 cases had completed immunohistochemical data for subtype classification analysis. All patients were evaluated by physical examinations to determine their T (tumor) and N (lymph node) stages. Routine investigations with BS, LUS and CXR were then carried out to detect subclinical metastases. Clinical staging was defined by American Joint Committee on Cancer (AJCC) System [21], based on the current use of subtype category [9-11]. Patients suspicious for bone metastases indicated by BS were confirmed by CT or MRI; suspicious for liver metastases indicated by LUS were confirmed by live dual phase scan CT; suspicious for lung metastases indicated by CXR were confirmed by chest CT or MRI. This retrospective study was approved by the institutional review board at the Third Affiliated Hospital of Harbin Medical University and conducted according to all current ethical guidelines.

### Statistics

All statistical analysis was conducted by SPSS software (version 15.0). Differences between categorical variables in metastatic events were evaluated by chi-square or Fisher’s exact tests. A p value of <0.05 was considered as statistically significant.

## Results

**Figure 1:**
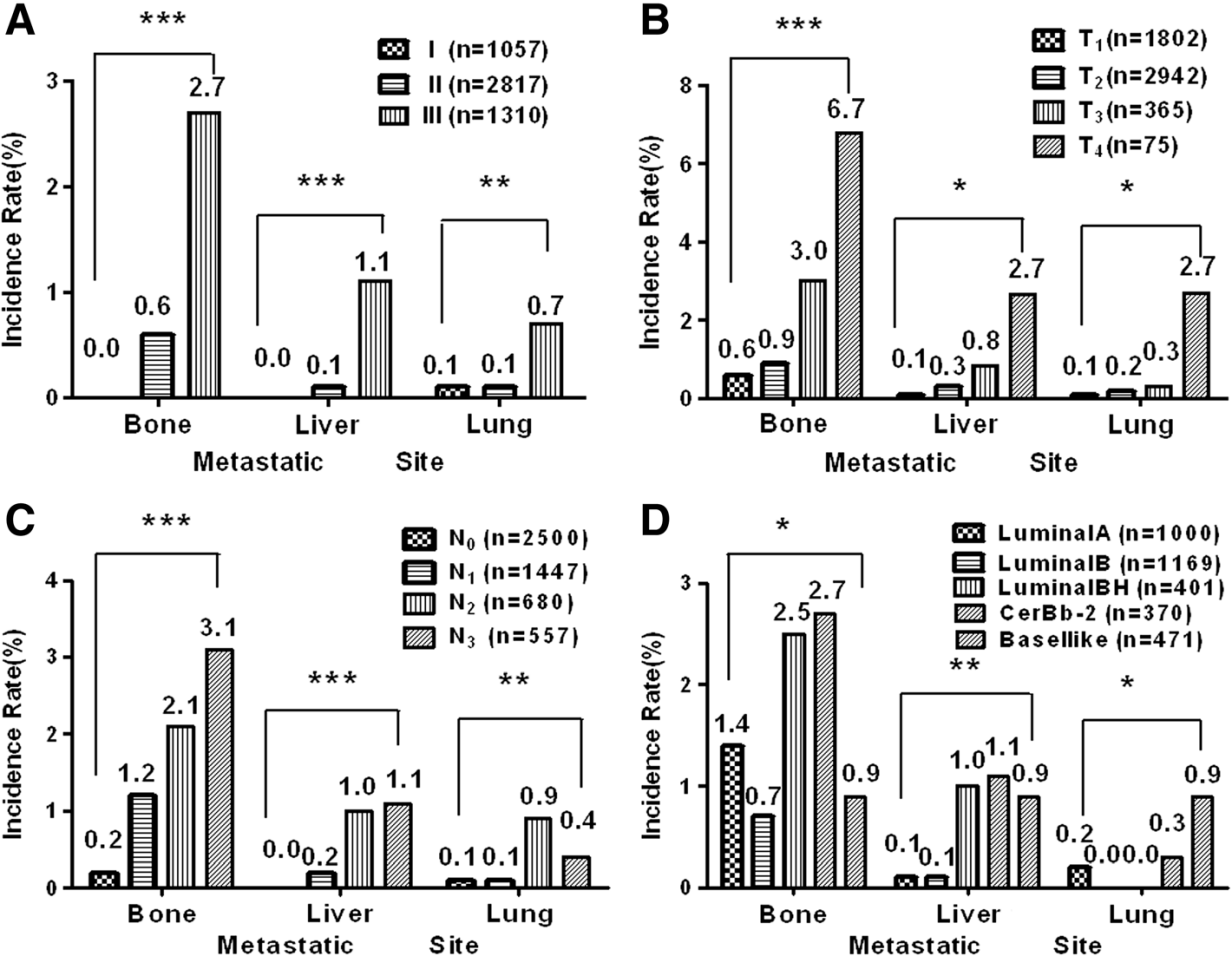
Frequency and distribution pattern of detectable metastatic diseases in bone, lung, and liver based on (A) clinical, (B) cT, (C) cN staging or (D) molecular subtypes.*P < 0.05, **P < 0.01, ***P < 0.001.

Metastases were more frequent in bone, than liver and lung as shown in Table 1 and Figure 1. For stage I, II, and III, bone metastasis rate was 0%, 0.6% and 2.7%, respectively (*P* < 0.01); liver metastasis rate was 0%, 0.1%, 1.1%, respectively, (*P* < 0.01); lung metastasis rate was 0.1%, 0.1%, 0.7%, respectively (*P* < 0.01).

**Table 1:** Detectable metastatic disease and clinical characteristics of breast cancer patients

		**Bone metastasis, n (%)**			**Liver metastasis, n (%)**			**Lung metastasis, n (%)**		
**Variables**	**No. of patients**	**(-)**	**(+)**	**P (*χ***^**2**^**)**	**(-)**	**(+)**	**P (*χ***^**2**^**)**	**(-)**	**(+)**	**P (*χ***^**2**^**)**
Age (years)										
≥ 35	3106	3064 (98.6)	42 (1.4)	1.000	3095 (99.6)	11 (0.4)	0.241	3100 (99.8)	6 (0.2)	0.481
< 35	305	301 (98.7)	4 (1.3)		302 (99.0)	3 (1.0)		304 (99.7)	1 (0.3)	
Histological differentiation										
I	411	406 (98.8)	5 (1.2)	0.649	409 (99.5)	2 (0.5)	0.95	410 (99.8)	1 (0.2)	0.839
II	2561	2528 (98.7)	33 (1.3)		2551 (99.6)	10 (0.4)		2556 (99.8)	5 (0.2)	
III	439	431 (98.2)	8 (1.8)		437 (99.5)	2 (0.5)		438 (99.8)	1 (0.2)	
cT stage										
cT1	1314	1304 (99.2)	10 (0.8)	0.000	1312 (99.8)	2 (0.2)	0.003	1313 (99.9)	1 (0.1)	0.04
cT2	1859	1833 (98.6)	26 (1.4)		1851 (98.6)	8 (1.4)		1855 (99.8)	4 (0.2)	
cT3	198	192 (97.0)	6 (3.0)		196 (98.9)	2 (1.0)		197 (99.5)	1 (0.5)	
cT4	40	36 (90.0)	4 (10.0)		38 (95.0)	2 (5.0)		39 (97.5)	1 (2.5)	
cN stage										
cN0	1592	1588 (99.7)	4 (0.3)	0.000	1592 (100.0)	0 (0.0)	0.000	1591 (99.9)	1 (0.0)	0.018
cN1	1044	1028 (98.5)	16 (1.5)		1042 (99.8)	2 (0.2)		1043 (99.9)	1 (0.2)	
cN2	462	449 (97.2)	13 (2.8)		456 (98.7)	6 (1.3)		459 (99.4)	3 (1.3)	
cN3	313	300 (95.8)	13 (4.2)		307 (98.1)	6 (1.9)		311 (99.4)	2 (1.9)	
ER status										
(-)	1317	1301 (98.8)	16 (1.2)	0.591	1307 (99.2)	10 (0.8)	0.011	1312 (99.6)	5 (0.4)	0.162
(+)	2094	2064 (98.6)	30 (1.4)		2090 (99.8)	4 (0.2)		2092 (99.9)	2 (0.1)	
PR status										
(-)	1131	1110 (98.4)	21 (1.6)	0.07	1122 (99.2)	9 (0.8)	0.028	1126 (99.6)	5 (0.8)	0.08
(+)	2280	2255 (98.8)	25 (1.2)		2275 (99.8)	5 (0.2)		2278 (99.9)	2 (0.1)	
HER2 status										
(-)	2640	2614 (99.0)	26 (1.0)	0.001	2634 (99.8)	6 (0.2)	0.006	2634 (99.8)	6 (0.2)	0.941
(+)	771	751 (97.4)	20 (2.6)		763 (99.0)	8 (0.1)		770 (99.9)	1 (0.1)	
Ki67 index										
≤ 14%	1331	1315 (98.8)	16 (0.2)	0.553	1325 (99.5)	6 (0.5)	0.768	1329 (98.8)	2 (1.2)	0.857
> 14%	2080	2050 (98.6)	30 (0.4)		2072 (99.6)	8 (0.4)		2075 (98.6)	5 (1.4)	

According to AJCC cT stage (tumor size), for patients with T1, T2, T3, and T4 disease, bone metastasis rate was 0.6%, 0.9%, 3.0%, 6.7% respectively (*P* < 0.01); live metastasis rate was 0.1%, 0.3%, 0.8%, 2.7%, respectively (*P* < 0.01); lung metastasis rate was 0.1%, 0.2%, 0.3%, 2.7%, respectively (*P* < 0.05).

According to AJCC cN stage (lymph node involvement), for patients with N0, N1, N2, and N3 disease, bone metastasis rate was 0.2%, 1.2%, 2.1%, and 3.1%, respectively (P < 0.01); liver metastasis rate was 0%,0.2%, 1.0%, and 1.1%, respectively (*P* < 0.01); lung metastasis rate was 0.1%, 0.1%, 0.9%, and 0.4%, respectively (*P* < 0.01).

Complete information on molecular subtype classification was available in 3411 patients. For patients defined as Luminal A, Luminal B, Luminal BH, HER2 overexpression, and Basal-like, bone metastasis rate was 1.4%, 0.7%, 2.5%, 2.7%, and 0.9%, respectively (P < 0.05); liver metastasis rate was 0.1%, 0.1%, 1.0%, 1.1%, and 0.9%, respectively (P < 0.01); lung metastasis rate was 0.2%, 0%, 0%, 0.3%, and 0.9%, respectively (P < 0.05).

Four factors (cT, cN, PR, and HER2) evaluated in the univariate analysis had significant influences on bone metastasis (*P* < 0.05). Five factors (cT, cN, ER, PR, and HER2) predicted liver metastasis (*P* < 0.05). Three factors (cT, cN, and PR) affected lung metastasis (*P* < 0.05).

## Discussion

In this study, we have observed different metastatic rates derived from molecular subtype classification and identified the predictors for bone, liver, lung metastasis in patients with newly diagnosed breast cancer without symptoms of distant metastases. Our findings suggest comprehensive BS, LUS and CXR tests are strongly recommended in those patients at diagnosis.

Incidence of bone metastasis varies widely in breast cancer. For patients with stage I, II, and III disease, bone metastasis rate is ranged from 0.1-6.8%, 0.8-8.8%, and 1.2-24.5% [1,2,4,22,23]. Those studies support that a BS is necessary for T3N1M0 and stage II/III patients with skeletal symptoms (such as pain and increased alkaline phosphatase). Consistently, we did not observe bone metastases in stage I patients. LUS is used to detect liver metastases, a relatively rare event in early-staged breast cancer patients. In our patient population, liver metastasis rate was increased with advance in clinical stage (0%, 0.07%, and 1.07% for stage I, II and III). Lung metastasis rate of stage I, II, and III disease could be ranged from 0–0.1%, 0.2-0.4%, 1.0-4.3%, respectively [2,4,23,24]. Although many anesthesiologists consider CXR as necessary prior to general anesthesia, there is no strong medical evidence to support that routine CXR is necessary before surgery for breast cancer patients without symptoms of distant metastases [24-26]. Our data showed lung metastasis rate of 0.1%, 0.1%, and 0.7% respectively, for patients with stage I, II, and III disease. Our results support that BS, LUS, and CXR are unnecessary for asymptomatic stage I and II patients as routine examinations.

Biological/pathological behaviors of breast cancer present race-/time-specificity [27]. For example, there are significant differences in tumor characteristics between patients in China and western countries [28]. The incidence of breast cancer metastasis identified by baseline staging was inconsistent [24] and very a few studies on Asian populations had been reported [29]. Undoubtedly, breast cancer is a group of heterogeneous diseases with substantial variation in both molecular and clinical characteristics. Rapid progress has been made in understanding the diversity of breast cancer, leading to a new molecular-driven integrated classification of breast cancer. The novel classification integrates molecular and clinical landscapes of breast cancer to define 5 clusters with distinct clinical outcomes and provide new insights into the management of the disease. Our findings have implications both for the individualization of therapy, bringing us a step closer to the realization of personalized medicine in breast cancer, but also provide a new evidence for exploring the underlying mechanisms of molecular subtypes [30].

Molecular subtype classification is a breakthrough in breast cancer research [9-11]. Different subtypes have different epidemiological risk factors, natural histories, and responses to treatment, which means that clinicians should consider distinct subtypes before selecting appropriate therapeutic strategies. Baseline staging tests after a new diagnosis of breast cancer based on subtype classification are debated. In our study, 3411 patients grouped into different subtypes showed different rates of bone, liver and lung metastasis. We also identified predictors for bone, liver and lung metastases such as cT, cN, ER, PR and HER2. Although these are not independent prognostic indicators, they are jointly determined the distribution of metastatic disease and can be used to refer to the baseline examination. The data indicate that patients with stage I breast cancer do not benefit from radiological staging for the detection of metastatic disease [18-20]. Preoperative BS, LUS and CXR should be considered for all of the stage III patients. For stage II patients, preoperative BS for Luminal BH, HER2 overexpression and Basal-like; preoperative LUS for Luminal BH, HER2 overexpression, and Basal-like; and CXR for Basal-like subtypes should be considered for early detection of distant metastases.

The five major molecular subtypes in breast cancer are different with regard to their ability to metastasize to distant organ(s), and share biological features and pathways with their preferred distant metastatic sites [31]. Previous studies of metastatic sites used postoperative follow-up data but we used the pre-treatment data in this study [32,33], which more accurately reflect the natural history of breast cancer, as well as the distribution and characteristics of metastatic sites in different subtypes without interference of treatments. Interestingly, our pattern of metastatic sites consistent with postoperative follow-up data, suggesting that metastases sites of breast cancer are dominated by the molecular subtypes [34], and less affected by treatments. Recent studies have found that molecular subtypes of breast cancer will change at relapse [35,36], while whether molecular subtypes modify the metastatic pattern should be explored in future. This retrospective study involves a large sample size compared with previous studies and investigates potential predictors for bone, liver, and lung metastasis of newly diagnosed asymptomatic breast cancer. The limitation is that all the clinicopathological variables were evaluated only by univariate models, due to inadequate of positive cases.

## Conclusions

In summary, rates of bone, liver, and lung metastasis showed significant differences between different subtypes of newly diagnosed breast cancer patients. Baseline staging tests BS, LUS and CXR after a new diagnosis of asymptomatic breast cancer are necessary based on subtype classification to avoid over– and under-treatment.

## Abbreviations

BS: Bone scanning; CT: Computed tomography; CXR: Chest radiography; ER: Estrogen receptor; HER2: Human epidermal growth factor receptor 2; LUS: Liver ultrasonography; MRI: Magnetic resonance imaging; PR: Progesterone receptor; TNBC: Triple negative breast cancer.

## Competing interests

There are no competing interests among the authors.

## Authors’ contributions

CX, SL, CY, LY, ZT, LQ, MQ, PH, ZY collected clinical information. CX, CY, SL, LY, ZT, CL and DX participated in the design of the study and performed the statistical analysis. CL and DX drafted the manuscript. All authors read and approved the final manuscript.
